# ﻿François Roffiaen's terrestrial and freshwater gastropod types in the collection of the Royal Belgian Institute of Natural Sciences

**DOI:** 10.3897/zookeys.1239.150840

**Published:** 2025-05-21

**Authors:** Rodrigo Brincalepe Salvador, Cedric d'Udekem d'Acoz, Maxim V. Vinarski, Yves Samyn, Barbara Mizumo Tomotani

**Affiliations:** 1 Zoology Unit, Finnish Museum of Natural History, University of Helsinki, Pohjoinen Rautatiekatu 13, 00100 Helsinki, Finland University of Helsinki Helsinki Finland; 2 Scientific Heritage Service, Partim Recent Invertebrates Collections, Royal Belgian Institute of Natural Sciences, Rue Vautier 29, 1000 Brussels, Belgium Partim Recent Invertebrates Collections, Royal Belgian Institute of Natural Sciences Brussels Belgium; 3 Saint-Petersburg State University, University Embankment 7/9, 199034 Saint-Petersburg, Russia Saint-Petersburg State University Saint-Petersburg Russia; 4 Arctic Chronobiology and Physiology Research Group, Department of Arctic and Marine Biology, UiT The Arctic University of Norway, Framstredt 41-42, 9019 Tromsø, Norway UiT The Arctic University of Norway Tromsø Norway

**Keywords:** Clausiliidae, Discidae, Helicidae, Lymnaeidae, Switzerland, Valvatidae, Viviparidae

## Abstract

Jean François Xavier Roffiaen (1820–1898) was a Belgian landscape painter with a profound interest in malacology. A founding member of the Société malacologique de Belgique, Roffiaen contributed several publications on molluscs. Among such studies, his 1868 paper on Swiss terrestrial and freshwater gastropods introduced 14 new taxa (species and varieties) belonging to the Clausiliidae, Discidae, Helicidae, Lymnaeidae, Valvatidae, and Viviparidae. However, Roffiaen’s malacological contributions largely faded from recognition, primarily due to the unknown whereabouts of his type material. This study revisits his work by identifying and analysing specimens from the Royal Belgian Institute of Natural Sciences (RBINS). Of the 14 taxa described by Roffiaen, type specimens for nine (including the two full species) have been recovered, enabling a reassessment of their taxonomic status as synonyms of better-known and widespread species. The serendipitous finding of these type specimens reaffirms the importance of maintaining museum collections, and the implementation of digitization programs to uncover/recover such “lost” information, enabling it to be made available to the scientific community at large.

## ﻿Introduction

Jean François Xavier Roffiaen (1820–1898) was a Belgian landscape painter, noted as one of the “minor masters” of the 19^th^ century (Hiernaux 1990b). He conducted his artistic studies first at the Académie de Namur and then at the Académie de Bruxelles, later travelling widely across Western Europe and developing a preference in portraying Alpine landscapes (Nève 1907; Hiernaux 1990a, 1990b, 1996; Hiernaux and Dewilde 2009). Roffiaen also developed a profound interest in natural sciences and, in particular, a closer interest in molluscs, in large part thanks to his close friend, the naturalist Jules Colbeau (Roffiaen 1881; Hiernaux 1989).

At the request of Colbeau, Roffiaen was one of the founders of the Société malacologique de Belgique in 1863, together with five other naturalists and mollusc enthusiasts (Lambotte 1863; Roffiaen 1881; Hiernaux 1989). He was an active member of the society, was its president for one year, and during this time his interest in molluscs grew. Over the years his enthusiasm enabled him to get several papers published in the “Annales de la Société malacologique de Belgique” (Nève 1907; Hiernaux 1989, 1990a; see the Appendix 1 for a list of Roffiaen’s publications, including short communications he presented during the society’s meetings). Roffiaen owned a collection of mollusc shells and kept live snails for observation and breeding (Hiernaux 1989, 1996). After the death of Colbeau in 1881, Roffiaen’s participation in the society, as well as his malacological publications, greatly decreased, though he remained a member and attended the meetings until 1897 (Hiernaux 1989). After his passing, the whereabouts of his conchological collection has been unknown.

As recognized by scholars studying the painter’s biography and artistic production (Hiernaux 1989; Hiernaux and Dewilde 2009), Roffiaen’s most important malacological paper is his study on Swiss gastropods, based on specimens that he collected during several (leisure) excursions to Switzerland, one of them (in 1852) together with Colbeau (Roffiaen 1868). This publication introduced 14 taxa as new to science (recognisable in that paper by the word “mihi”), including both species and infraspecific taxa (regarded as varieties in the original paper), of freshwater and terrestrial snails. Roffiaen’s terrestrial taxa were never again featured or reassessed and are absent from the mainstream European or Swiss malacological literature such as Turner et al. (1998) and Welter-Schultes (2012).

Most of the freshwater taxa were “luckier”, with lymnaeids being cited in the studies of Servain (1882), Hubendick (1951) and Vinarski (2024). Of special interest is a work on the taxonomy of the family Lymnaeidae by Kruglov and Starobogatov (1993). These authors resurrected one of Roffiaen’s taxa, Limnaeatruncatulavar.subangulata, and considered it a “good” species referred to as Lymnaea (Galba) subangulata. However, those authors did not examine the type series of Roffiaen’s taxon, and thus, the identity of this purported species has remained obscure, being largely considered a synonym of the nominate taxon.

As shown, Roffiaen’s taxa remained largely unknown and undocumented after the original work. The prime reason for which is likely the fact that the whereabouts of the type material remained unknown and, thus, has never been critically re-studied since the original publication. This now changed, as the type specimens of the majority of Roffiaen’s taxa have recently been found in the collection of the Royal Belgian Institute of Natural Sciences (RBINS; Brussels, Belgium). We illustrate those specimens here to allow a contemporary examination of Roffiaen’s taxa, enabling us to include them in current understanding of the gastropod groups involved and present a discussion regarding their taxonomic status.

## ﻿Material and methods

### ﻿List of taxa described by Roffiaen

Roffiaen (1868) described seven new freshwater gastropod taxa, one being a species (*Valvatacolbeaui* Roffiaen, 1868) and six being varieties: Paludinacontectavar.emiliana Roffiaen, 1868, Limnaeaperegravar.pulchella Roffiaen, 1868, Limnaeastagnalisvar.productissima Roffiaen, 1868, Limnaeatruncatulavar.subangulata Roffiaen, 1868, Limnaeapalustrisvar.fallaciosa Roffiaen, 1868, and Limnaeapalustrisvar.pellucida Roffiaen, 1868.

Roffiaen (1868) also described seven terrestrial gastropod taxa, consisting of one species (*Clausiliaweyersi* Roffiaen, 1868) and six varieties: Clausiliaplicatavar.elongata Roffiaen, 1868, Clausiliaplicatulavar.albinos Roffiaen, 1868, Helixruderatavar.viridana Roffiaen, 1868, Helixarbustorumvar.trochoidalis Roffiaen, 1868, Helixarbustorumvar.marmorata Roffiaen, 1868, and Helixarbustorumvar.icterica Roffiaen, 1868.

### ﻿Material examined

Given that Roffiaen’s (1868) taxa were largely forgotten in previous literature, we considered it worthwhile to study each specimen identified with his taxa’s names. Therefore, we thoroughly screened the historical collections of the RBINS to recover as many as possible of Roffiaen’s specimens.

Assessment of the type status of each specimen lot was assured by assessing multiple lines of evidence: coincidence of collection locality; coincidence of collector; presence of the word “type” or similar on the label (particularly the older labels); comparison of specimens to published illustrations in Roffiaen (1868); indication that the lot belonged to either Roffiaen’s own collection, to that of his close associate Jules Colbeau (who accompanied him on the 1852 collecting trip that resulted in the paper; Roffiaen 1868: 66), or to that of his contemporary Hugo de Cort (Coan and Kabat 2023) (labels in the RBINS collection indicate that de Cort acquired specimens from Roffiaen’s collection). The rationale for our assessment of each taxon is given case-by-case in the Systematics section below.

Specimens were photographed using, for the largest specimens, a Canon EOS 60D camera equipped with an EF-S35mm f/2.8 MACRO IS STM lens and producing images of 5184 × 3456 pixels, and, for the smallest specimens, a Canon EOS 6D Mark II camera equipped with an MP-E65mm f/2.8 1x5 Macro Photo lens and producing 6240 × 4160 pixels. The cameras were mounted on a “Cognisys StackShot Macro Rail Package Automated Stacking Image Capture for Stacking” adapted to a VELBON Sherpa 600R tripod, fitted with a VELBON PH-157Q handle. Specimens were photographed without flash, in a PULUZ light box, raised on a polystyrene block, either on a sheet of black velvet or on black modelling clay for optimal positioning. Individual photographs were calibrated and generated using Helicon Focus software (Helicon Soft, Ukraine). Between 15 and 40 photos were taken at predetermined intervals. The photographs were stacked using Zerene Stacker software (Zerene Systems, USA). Figures were created using the software Corel PHOTO-PAINT (Alludo, Canada).

MicroCT scans were taken using the EasyTom 150 - RX Solution scanner (RX Solutions, France). The source was shielded by an aluminium filter, the voltage was 110 kV and the photographs were taken in Small Focus Mode (1440 projections per rotation; simple scan or stack 2x). Exposure varied from one specimen to another, generally with a digital gain of 2x. In general, 6 averages were taken. X-Act (RX Solutions) was used to generate slices and an automatic 3D model, which was then cleaned and smoothed with GOM Inspect. In some cases, rendering images were obtained with Dragonfly ORS (Comet Technologies, Canada) and snapshots were exported.

### ﻿Taxonomy

The taxonomic status of Roffiaen’s taxa was assessed by using current taxonomic literature, published photographs of specimens, and voucher specimens in the collection of the Finnish Museum of Natural History (MZH, Helsinki, Finland). The exceptions are the Lymnaeidae taxa, for which the works of Hubendick (1951) and Vinarski (2024) were followed.

For the varieties described by Roffiaen (1868), it is generally straightforward to assess their status as junior synonyms of the nominate species, considering the present knowledge on conchological variation and geographic distribution of those taxa compared to when they were first described. Roffiaen’s new valvatid species, *Valvatacolbeaui*, is a little more problematic, as there is a lack of information on conchological variation and genetic data for this taxon. We discuss each case in turn in the Systematics session below, but for some we are only able to provide tentative assessments.

## ﻿Results

Of Roffiaen’s 14 new taxa, we found potential type specimens (including syntypes and holotypes) of nine taxa, namely: Paludinacontectavar.emiliana, *Valvatacolbeaui*, Lymnaeapalustrisvar.fallaciosa, Lymnaeapalustrisvar.pellucida, Lymnaeaperegravar.pulchella, Clausiliaplicatavar.elongata, *Clausiliaweyersi*, Helixarbustorumvar.icterica, and Helixarbustorumvar.trochoidalis. Topotypes of Lymnaeastagnalisvar.productissima were found, but these were deemed not to be type specimens per se. For four taxa, no specimens (type or otherwise) were found: Lymnaeatruncatulavar.subangulata, Clausiliaplicatulavar.albinos, Helixruderatavar.viridana, and Helixarbustorumvar.marmorata. All recovered material consists of dry shells only, no preserved animals (in ethanol or other preservation fluid) have been found.

Below we list each of Roffiaen’s taxa, the recovered type specimens (when available), and assess their taxonomic and nomenclatural status. The list is arranged systematically and within each family alphabetically on the lowest taxon epithet. The current taxonomic status of each taxon is provided and, when necessary, accompanied by a brief discussion. Images of the labels and at least one syntype of each species are provided, when available. Further images and 3D models are available through RBINS Collections portal (https://collections.naturalsciences.be/).

### ﻿Systematics

#### ﻿ARCHITAENIOGLOSSA

**Superfamily Viviparoidea**

**Family Viviparidae**

##### ﻿ Paludina contecta var. emiliana

Taxon classificationAnimaliaArchitaenioglossaViviparidae

Roffiaen, 1868

D72C5B6C-A24A-5B27-9D70-E291162211DA

Fig. 1A

Paludina contecta var. emiliana Roffiaen, 1868: 80.

###### Type material.

RBINS I.G.34957/MT.2029: 1 holotype, ex Roffiaen coll.

###### Type locality.

Italy: Arona, Lago Maggiore, Italian shore of the lake. “[…] Lac Majeur, à Arona, sur la rive italienne” (Roffiaen 1868: 80).

###### Taxonomic status.

Junior synonym of *Viviparuscontectus* (Millet, 1813).

###### Discussion.

According to the specimen’s label, it stems from the collection of Roffiaen, it is from the type locality and the word “type” is written on it in the same handwriting. Considering that the description of Roffiaen (1868) was based on a single shell, the present specimen can be considered the holotype.

**Figure 1.**
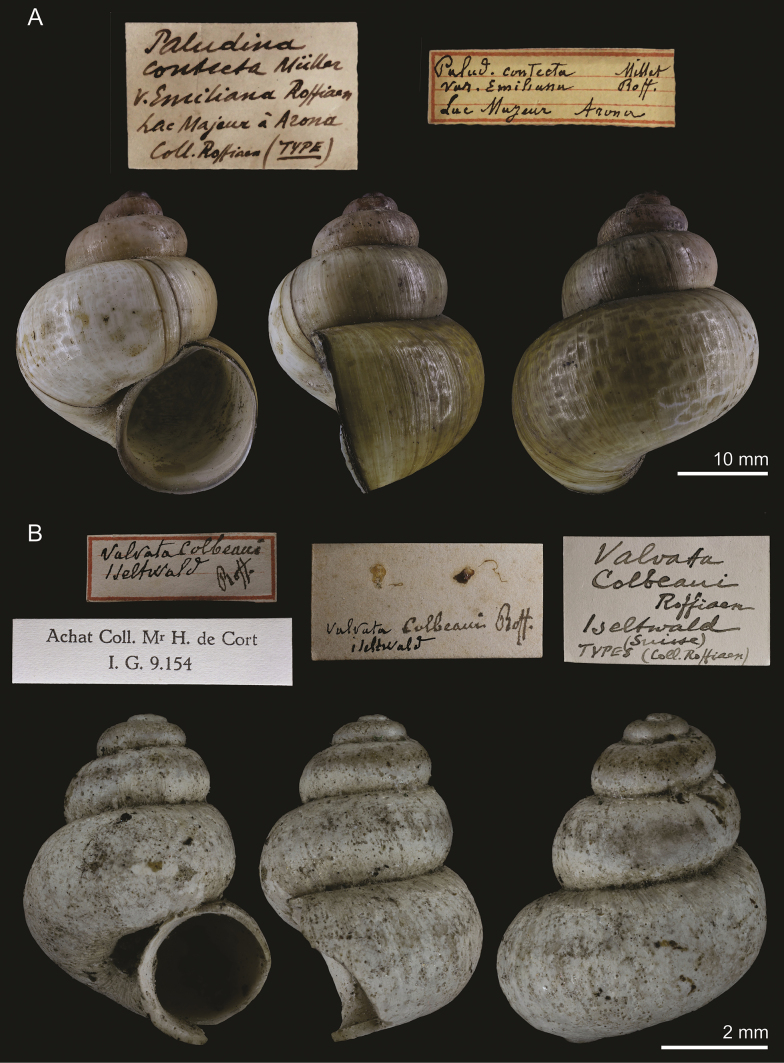
**A** holotype of Paludinacontectavar.emiliana Roffiaen, 1868, RBINS I.G.34957/MT.2029 **B** syntype of *Valvatacolbeaui* Roffiaen, 1868, RBINS I.G.9154/MT.2041. Labels not to scale with shells.

This taxon was distinguished by Roffiaen (1868) as lacking coloured spiral bands and by the first whorls being purplish. Both colour variations are observed in *Viviparuscontectus*, a species that is known from the region, and the shell shape of the present specimen (Fig. 1A) is likewise consistent with that species, including the “hammered” markings on the teleoconch (Welter-Schultes 2012; Rowson et al. 2021). Nevertheless, one key diagnostic feature of *V.contectus*, the sharply pointed tip of the spire (Rowson et al. 2021), is not observed in the present specimen. Still, *V.contectus* is known for conchological variation across its distribution (Welter-Schultes 2012) and, thus, *Paludinacontectaemiliana* is here considered its junior synonym.

#### ﻿“LOWER HETEROBRANCHIA”

**Superfamily Valvatoidea**

**Family Valvatidae**

##### ﻿ Valvata colbeaui

Taxon classificationAnimaliaArchitaenioglossaValvatidae

Roffiaen, 1868

5BC24B03-AB65-5322-946A-4E1501D8C77C

Fig. 1B

Valvata colbeaui Roffiaen, 1868: 81, pl. 1, fig. 1.

###### Type material.

RBINS I.G.9154/MT.2041: 2 syntypes, ex Roffiaen coll., ex de Cort coll.

###### Type locality.

Switzerland: Iseltwalt, Lake Brienz (Brienzersee). “Lac de Brientz à Iseltwalt” (Roffiaen 1868: 81).

###### Taxonomic status.

Junior synonym of *Valvataantiqua* Morris, 1838 (see below).

###### Discussion.

According to the label, the specimens stem from the collection of Roffiaen, they are from the type locality (Iseltwalt) and are labelled “types”. Thus, they can be considered syntypes. It is also worthwhile to note that the lot first became part of de Cort’s collection before being acquired by the RBINS.

Roffiaen’s shells correspond reasonably well to *Valvataantiqua* Morris, 1838, a morphospecies for which genetic data remains unavailable and, consequently, a variety of opinions on its taxonomic status and rank exists among malacologists. It is sometimes regarded as merely an infraspecific morph of *V.piscinalis* thought to develop in large lakes, including the Alpine lakes (Falniowski 1989; Piechocki and Wawrzyniak-Wydrowska 2016). Falniowski (1989: 73) considered this morph *Valvatapiscinalis* f. *antiquacolbeaui*, an endemic to Lake Geneva. Glöer (2002) ranked it as a subspecies of *V.piscinalis*, but this solution is untenable since the ranges of *V.piscinalispiscinalis* and *V.piscinalisantiqua* broadly overlap. The latest publications tend to accept *V.antiqua* as a species, distinct from *V.piscinalis* (Vinarski and Kantor 2016; Glöer 2019), although a DNA assessment of this insufficiently studied snail is urgently needed.

*Valvatapiscinalis* (O.F. Müller, 1774) is a widespread species in Europe, and while it more usually displays a wide shell with an ample body whorl and aperture, more trochoid forms with comparatively smaller apertures are also known (Turner et al. 1998; Glöer 2002, 2019; Vinarski et al. 2013). The present specimens at first seem to fit within the range of conchological variation displayed by that species, though they are smaller at the same number of whorls. The largest specimen available (Fig. 1B), which is the most similar to the published illustration (Roffiaen 1868: pl. 1, fig. 1), has a more strongly trochoid shell, with narrower and less bulging whorls.

##### HYGROPHILA

**Superfamily Lymnaeoidea**

**Family Lymnaeidae**

###### ﻿ Lymnaea palustris var. fallaciosa

Taxon classificationAnimaliaArchitaenioglossaLymnaeidae

Roffiaen, 1868

0F5EF3EE-4431-54AE-9BEE-D6112F2E0C8B

Fig. 2A

Limnæa palustris var. fallaciosa Roffiaen, 1868: 78, pl. 1, fig. 6.

####### Type material.

RBINS I.G.7065/MT.4174: 3 syntypes, ex Colbeau coll. RBINS I.G.28242/HIST.180: 6 possible syntypes. RBINS I.G.7065/HIST.181: 1 possible syntype.

####### Type locality.

Switzerland: Brunnen, along road to Schwyz. “[…] Brunnen, dans un fossé de la plaine, longeant la route de Schwyz” (Roffiaen 1868: 79).

####### Taxonomic status.

Junior synonym of *Stagnicolapalustris* (O.F. Müller, 1774) (cf. Servain 1882; Hubendick 1951; Vinarski 2024).

####### Discussion.

Lot RBINS I.G.7065/MT.4174 stems from the collection of Jules Colbeau, comes from the type locality (Brunnen), and the specimens are a good match to the published illustration (Roffiaen 1868: pl. 1, fig. 6); thus, the three specimens it contains are here considered syntypes.

Lot RBINS I.G.28242/HIST.180 stems from the type locality but bear no other information regarding their former owners. The six specimens match morphologically the syntypes in the previous lot and, thus, are considered here as possible syntypes. Similarly, lot RBINS I.G.7065/HIST.181 has 1 specimen from Colbeau’s collection whose location is given as “Schwyz” on the label, together with an indication that this specimen belonged to the study of Roffiaen. While the type locality of the present taxon could be generously interpreted as containing Schwyz, we deem it safer to consider the present specimen as a possible syntype.

**Figure 2.**
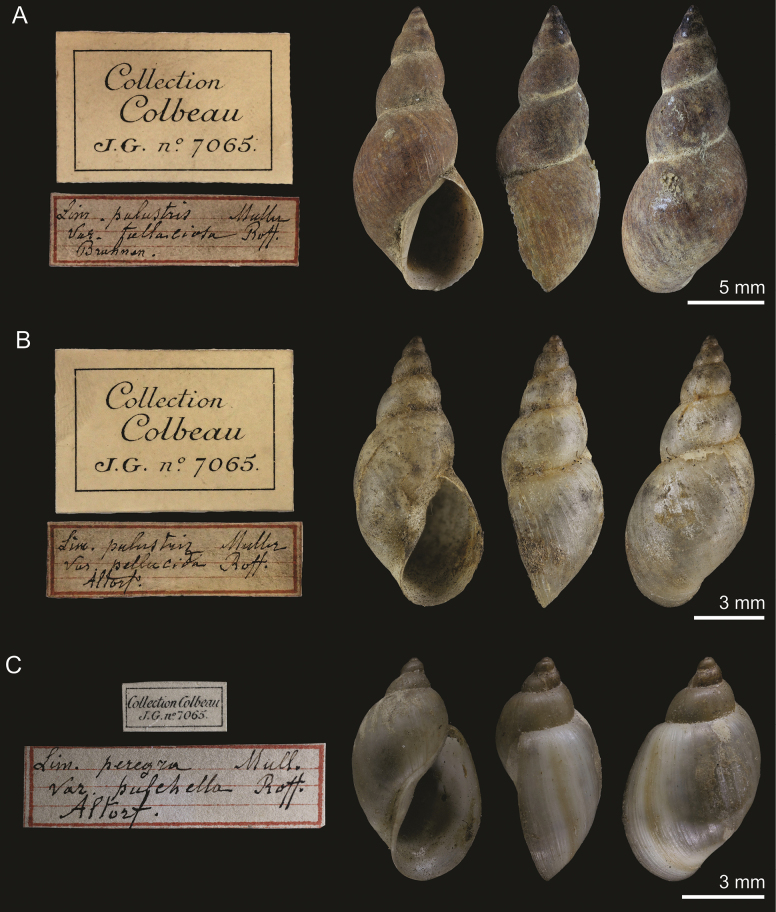
**A** syntype of Lymnaeapalustrisvar.fallaciosa Roffiaen, 1868, RBINS I.G.7065/MT.4174 **B** holotype of Lymnaeapalustrisvar.pellucida Roffiaen, 1868, RBINS I.G.7065/MT.4175 **C** syntype of Lymnaeaperegravar.pulchella Roffiaen, 1868, RBINS I.G.7065/MT.4173. Labels not to scale with shells.

Though conchologically, this variety corresponds well to a widespread *Stagnicolapalustris*, there is a possibility that Roffiaen’s shells could belong to *S.fuscus* (C. Pfeiffer, 1821) instead. The latter can only be distinguished with certainty from *S.palustris* by anatomical and/or genetic investigations (Welter-Schultes 2012; Glöer 2019). The range of *S.fuscus* covers much of central and western Europe, including Switzerland (Welter-Schultes 2012).

###### ﻿ Lymnaea palustris var. pellucida

Taxon classificationAnimaliaArchitaenioglossaLymnaeidae

Roffiaen, 1868

A1C30208-37EF-5CBD-9C70-8A8B0D4CBB55

Fig. 2B

Limnæa palustris var. pellucida Roffiaen, 1868: 79, pl. 1, fig. 7.

####### Type material.

RBINS I.G.7065/MT.4175: 1 holotype, ex Colbeau coll.

####### Type locality.

Switzerland: Altdorf, in a ditch on the plains towards Seedorf. “[…] dans un fossé de la plaine d’Altorf, vers Seedorf (M. Colbeau)” (Roffiaen 1868: 79).

####### Taxonomic status.

Junior synonym of *Stagnicolapalustris* (O.F. Müller, 1774) (cf. Servain 1882; Hubendick 1951; Vinarski 2024).

####### Discussion.

Specimen RBINS I.G.7065/MT.4175 stems from the collection of Jules Colbeau, comes from the type locality (Altdorf, spelled “Altorf” on the label as in the original publication) and is a good match to the published illustration (Roffiaen 1868: pl. 1, fig. 7). Considering the original description was written using a single individual, the present specimen can thus be considered the holotype.

A possibility that the holotype could belong to *S.fuscus* cannot be rejected (see the discussion of L.palustrisvar.fallaciosa above).

###### ﻿ Lymnaea stagnalis var. productissima

Taxon classificationAnimaliaArchitaenioglossaLymnaeidae

Roffiaen, 1868

D8A90467-0E07-5E74-9510-8EE167D09A7C

Limnæa stagnalis var. productissima Roffiaen, 1868: 78: pl. 1, fig. 5.

####### Type material.

Not located.

####### Type locality.

Switzerland: Magadino, Lago Maggiore. “Lac Majeur à Magadino” (Roffiaen 1868: 78).

####### Taxonomic status.

Junior synonym of *Lymnaeastagnalis* (Linnaeus, 1758) sensu lato (cf. Hubendick 1951; Vinarski 2024).

####### Discussion.

There is only one lot (RBINS I.G.7065/HIST.372, containing 7 shells) in the collection identified as Roffiaen’s taxon, collected in the type locality (“Lac Majeur Magadino” on the label). There is unfortunately no further collection data for this specimen lot, so it cannot be ascertained whether these shells came from Roffiaen. In any event, while the specimens do represent *Lymnaeastagnalis*, they are only about half the size of Roffiaen’s illustrated adult specimen (Roffiaen 1868: pl. 1, fig. 5). Consequently, the shell morphology of these specimens does not correspond with Roffiaen’s diagnosis (“*Coguille très-allongée et efilée à dernier tour moins ventru que chez le type*”, i.e., very elongated and tapered shell with body whorl less rounded than in the type; Roffiaen 1868: 78). Thus, these specimens are likely not types of *Lymnaeastagnalisproductissima*. Being collected from a large lake, the shells most probably belong to a “lacustrine” morph of *L.stagnalis* sensu lato, which is characterized by reduced body size and shortened spire (see Geyer 1929; Hubendick 1951).

###### ﻿ Lymnaea peregra var. pulchella

Taxon classificationAnimaliaArchitaenioglossaLymnaeidae

Roffiaen, 1868

0B12AA70-BC9C-5458-8FF9-3A760016471D

Fig. 2C

Limnæa peregra var. pulchella Roffiaen, 1868: 77, pl. 1, fig. 8.

####### Type material.

RBINS I.G.7065/MT.4173: 3 syntypes, ex Colbeau coll.

####### Type locality.

Switzerland: Altdorf, in a ditch on the plains towards Seedorf. “[…] dans un fossé de la plaine d’Altorf vers Seedorf (M. Colbeau)” (Roffiaen 1868: 77).

####### Taxonomic status.

Junior synonym of *Peregrianaperegra* (O.F. Müller, 1774) (cf. Hubendick 1951; Vinarski 2024).

####### Discussion.

The present lot stems from the collection of Jules Colbeau, it comes from the type locality (Altdorf, spelled “Altorf” on label as in the original publication), and the specimens are a good match to the published illustration (Roffiaen 1868: pl. 1, fig. 8); the three specimens it contains are here considered syntypes.

###### ﻿ Lymnaea truncatula var. subangulata

Taxon classificationAnimaliaArchitaenioglossaLymnaeidae

Roffiaen, 1868

1B63B095-42E9-51EB-ABC0-8905F39D01F5

Limnæa truncatula var. subangulata Roffiaen, 1868: 78, pl. 1, fig. 9.

####### Type material.

Not located.

####### Type locality.

Switzerland: Altdorf, in a ditch on the plains towards Seedorf. “[…] prés d’Altorf, dans un fossé de la plaine vers Seedorf (M. Colbeau)” (Roffiaen 1868: 78).

####### Taxonomic status.

Junior synonym of *Galbatruncatula* (O.F. Müller, 1774) (cf. Hubendick 1951; Vinarski 2024).

####### Discussion.

No specimens belonging to this taxon were found. The original description mentions a few specimens and describe them as being “contorted and misshapen” (“contourné et difforme”; Roffiaen 1868: 78) and the accompanying illustrations confirm that description. Likely, these specimens are individuals of *Galbatruncatula* that experienced some problems during growth (e.g., shell breakage and consequent scar and irregular shell growth).

A species that has been named Lymnaea (Galba) subangulata in the Russian literature (Kruglov and Starobogatov 1993; Kruglov 2005; Vinarski and Kantor 2016) differs from Roffiaen’s Limnæatruncatulavar.subangulata. The shell of the former is not “contorted and misshapen” and represents, possibly, an intraspecific morph of *G.truncatula* characterized by a rounded shell with small spire and somewhat inflated body whorl.

##### STYLOMMATOPHORA

**Superfamily Clausilioidea**

**Family Clausiliidae**

###### ﻿ Clausilia plicatula var. albinos

Taxon classificationAnimaliaStylommatophoraClausiliidae

Roffiaen, 1868

2EEF7D40-1C85-5896-AB5D-AC575189742F

Clausilia plicatula var. albinos Roffiaen, 1868: 76.

####### Type material.

Single specimen, at present not located.

####### Type locality.

Switzerland: Iseltwald.

####### Taxonomic status.

Junior synonym of *Macrogastraplicatulaplicatula* (Draparnaud, 1801).

####### Discussion.

No specimens belonging to this taxon were found. The original description mentioned a single shell, whitish and transparent (hence, “albinos”), as belonging to this variety, while commenting that the nominate form is common and very variable (Roffiaen 1868: 76). Thus, this variety based on an albino specimen (or otherwise presenting partial or total loss of pigments) can be considered synonymous with the nominate form. *Macrogastraplicatula* is widespread in Europe, including many records in the area encompassing Iseltwald representing *M.p.plicatula* (Turner et al. 1998; Nordsieck 2006).

###### ﻿ Clausilia plicata var. elongata

Taxon classificationAnimaliaStylommatophoraClausiliidae

Roffiaen, 1868

444E414F-87A2-5C0B-ACB0-3BA9F379AF08

Fig. 3A

Clausilia plicata var. elongata Roffiaen, 1868: 75, pl. 1, fig. 4.

####### Type material.

RBINS I.G.9154/MT.1771: 3 possible syntypes, ex de Cort coll.

####### Type locality.

Switzerland: Andeer.

####### Taxonomic status.

Junior synonym of *Laciniariaplicataplicata* (Draparnaud, 1801).

####### Discussion.

According to the specimens’ labels, they were collected at the type locality Andeer and stem from the collection of Hugo de Cort. Furthermore, one of the labels states “types”, but there is no mention of Roffiaen or his collection. Thus, the present specimens are here considered as possible syntypes.

**Figure 3.**
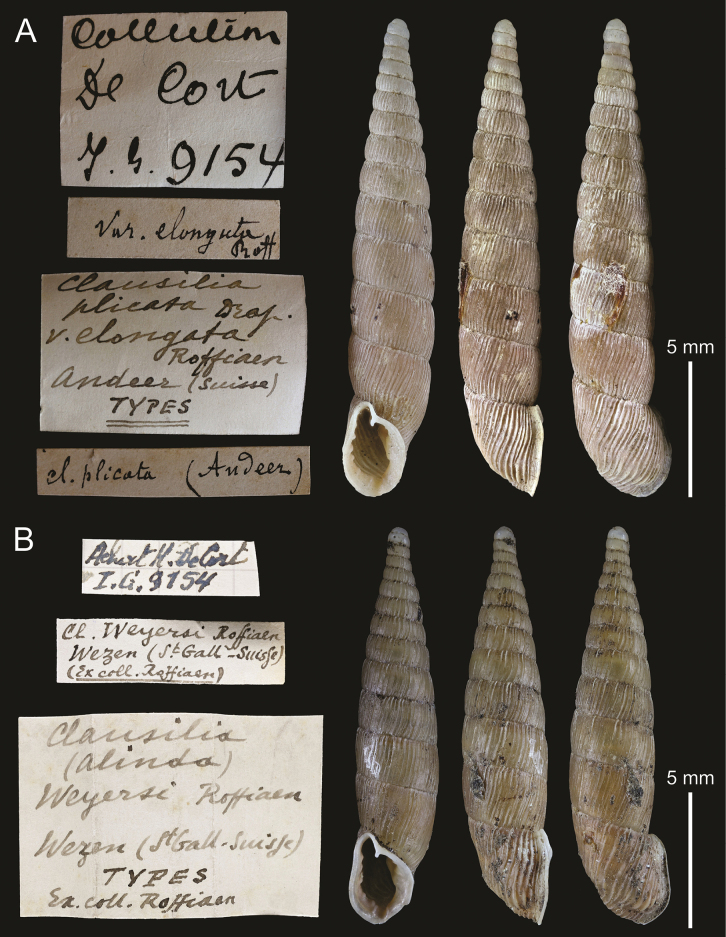
**A** possible syntype of Clausiliaplicatavar.elongata Roffiaen, 1868, RBINS I.G.9154/MT.1771 **B** syntype of *Clausiliaweyersi* Roffiaen, 1868, RBINS I.G.9154/MT.1773. Labels not to scale with shells.

*Laciniariaplicataplicata* is widely distributed in Central Europe, including Switzerland (Turner et al. 1998; Nordsieck 2008). The elongated spire of Roffiaen’s variety is well within the known variation in shell shape and size known in *L.p.plicata* (Nordsieck 2008).

###### ﻿ Clausilia weyersi

Taxon classificationAnimaliaStylommatophoraClausiliidae

Roffiaen, 1868

3FB19793-6A49-5DC1-9DBB-8DC6D08CE0FB

Fig. 3B , Suppl. material 1

Clausilia weyersi Roffiaen, 1868: 75, pl. 1, fig. 3.

####### Type material.

RBINS I.G.9154/MT.1773: 2 syntypes, ex Roffiaen coll., ex de Cort coll. RBINS I.G.9154/HIST.2486: 29 possible syntypes, ex de Cort coll.

####### Type locality.

Switzerland: Weesen. “Wesen” (Roffiaen 1868: 75).

####### Taxonomic status.

Junior synonym of *Laciniariaplicataplicata* (Draparnaud, 1801).

####### Discussion.

Lot RBINS I.G.9154/MT.1773 comes from the type locality, belonged to Roffiaen’s collection, and bears the writing “types”; its specimens are considered syntypes. Lot RBINS I.G.9154/HIST.2486, however, while coming from the type locality, has no indication to have belonged to Roffiaen’s collection or of being types, though they belonged to de Cort’s collection. Therefore, the later specimens are considered possible syntypes.

*Clausiliaweyersi* bears close resemblance to typical specimens of *Laciniariaplicataplicata*, both regarding shell shape, size and apertural barriers (Nordsieck 2008; see Suppl. material 1 for CT scan snapshots of internal structures of shell). It can thus, be considered a junior synonym of the latter.

##### Superfamily Discoidea

**Family Discidae**

###### ﻿ Helix ruderata var. viridana

Taxon classificationAnimaliaStylommatophoraDiscidae

Roffiaen, 1868

1B0E640C-54CB-57CE-B3D9-E3320CEA699E

Helix ruderata var. viridana Roffiaen, 1868: 68.

####### Type material.

Not located.

####### Type locality.

Switzerland: Handeck (Handegg). “La Handeck” (Roffiaen 1868: 68).

####### Taxonomic status.

Junior synonym of *Discusruderatus* (Hartmann, 1821).

####### Discussion.

This variety was described to allocate specimens for which the single conchological character noted by Roffiaen (1868) was the “pale greenish colour”. The shell of *Discusruderatus* is known to typically to be light brown to reddish brown (Welter-Schultes 2012), though paler yellowish and greenish brown specimens are also common (Kerney et al. 1979; Wiese 2014). Furthermore, the type locality of this variety is within the known distribution of *D.ruderatus* in Switzerland (Turner et al. 1998), and Roffiaen (1868) himself mentioned “typical” *D.ruderatus* occurring in the same place. Thus, even without access to type material, it can be surmised that *Helixruderataviridana* is very likely a junior synonym of *D.ruderatus*.

##### Superfamily Helicoidea

**Family Helicidae**

###### ﻿ Helix arbustorum var. icterica

Taxon classificationAnimaliaStylommatophoraHelicidae

Roffiaen, 1868

3F582E7A-F3FC-584A-B386-086C250D300F

Fig. 4A

Helix arbustorum var. icterica Roffiaen, 1868: 70.

####### Type material.

RBINS I.G.7065/MT.3421: 1 syntype from Weesen, ex Colbeau coll. (nr. 7065).

####### Type locality.

Switzerland: Tamina, Weesen, Iseltwald, Meiringen, Sarnen. “Gorge de la Tamina, Wesen, Iseltwald, Meyringen, Sarnen” (Roffiaen 1868: 70).

####### Taxonomic status.

Junior synonym of *Ariantaarbustorum* (Linnaeus, 1758).

####### Discussion.

The present specimen stems from the collection of Jules Colbeau, is from one of the localities that constitute the taxon’s type locality as mentioned by Roffiaen (1868) and the label bears the writing “type”. Thus, it can be considered a syntype.

**Figure 4.**
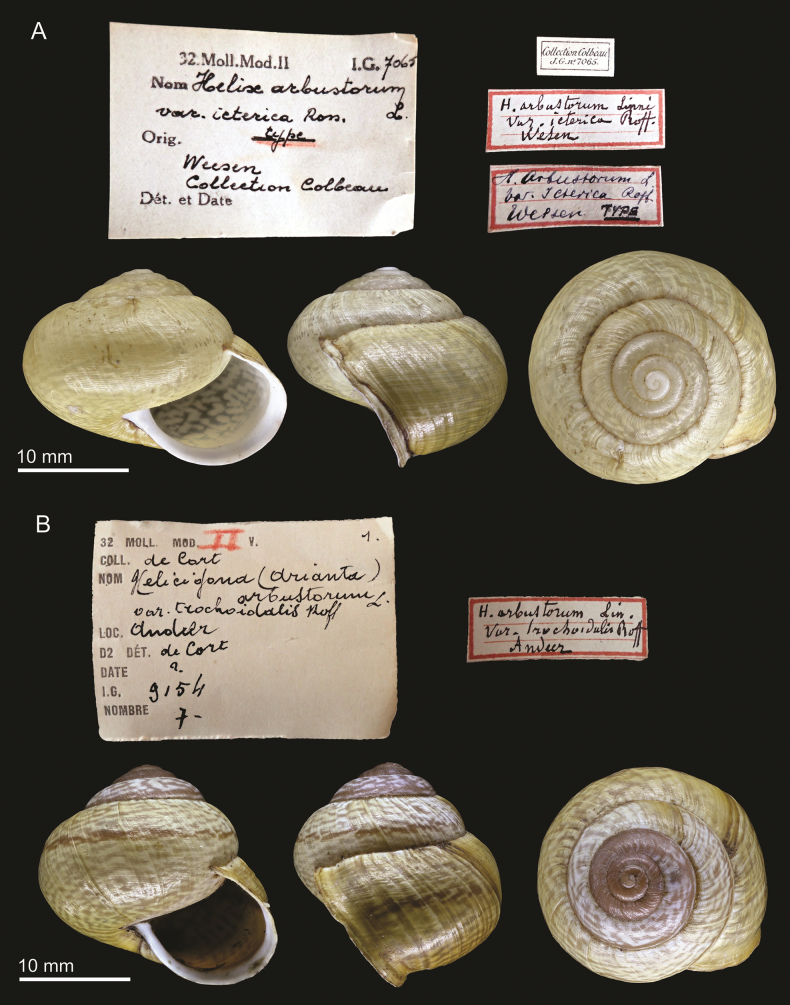
**A** syntype of Helixarbustorumvar.icterica Roffiaen, 1868, RBINS I.G.7065/MT.3421 **B** possible syntype of Helixarbustorumvar.trochoidalis Roffiaen, 1868, I.G.9154/MT.4172. Labels not to scale with shells

This variety was described to denote specimens lacking the typical colour pattern of nominate *A.arbustorum*, consisting instead of entirely pale-yellow shells. Roffiaen also mentioned that such specimens were “not common but not rare either” (Roffiaen 1868: 70). This type of colouration is often seen in some members of this species across its range, consistent with natural interspecific phenotypic variation and sometimes is more common at some localities than others (e.g., Grime and Blythe 1969; Parkin 1971; Arter 1990; Cameron and Riley 2008; von Proschwitz et al. 2023). Thus, *A.A.icterica* is here considered synonymous with *A.arbustorum*. The variability in shell shape, size and colour of *A.arbustorum* has led to many forms, varieties, and subspecies to be described in the past centuries, which have almost always been considered junior synonyms (e.g., Taylor 1914).

###### ﻿ Helix arbustorum var. marmorata

Taxon classificationAnimaliaStylommatophoraHelicidae

Roffiaen, 1868

3FF1EC14-161B-5577-9931-BC58DDB06A95

Helix arbustorum var. marmorata Roffiaen, 1868: 70.

####### Type material.

Not located.

####### Type locality.

Switzerland: Weesen. “Se trouve en diverses localités, n’est pas rare à Wesen” (Roffiaen, 1868: 70).

####### Taxonomic status.

Junior synonym of *Ariantaarbustorum* (Linnaeus, 1758).

####### Discussion.

This variety was described to denote specimens lacking the dark spiral bands seen on the shells of typical *Ariantaarbustorum*. As for the case of the yellow shells of the variety *icterica* above, this is part of the common colour polymorphism known in *A.arbustorum* (Cook and King 1966; Grime and Blythe 1969; Burla and Stahel 1983; Burla 1984; von Proschwitz et al. 2023). Thus, even without having access to type material, Roffiaen’s (1868) description, along with current knowledge on *A.arbustorum*, is sufficient to surmise that *A.A.marmorata* is synonymous with *A.arbustorum*.

###### ﻿ Helix arbustorum var. trochoidalis

Taxon classificationAnimaliaStylommatophoraHelicidae

Roffiaen, 1868

9EBC9B42-B937-5C5B-982E-E560972BB696

Fig. 4B

Helix arbustorum var. trochoidalis Roffiaen, 1868: 69, pl. 1, fig. 2.

####### Type material.

RBINS I.G.9154/MT.4172 and I.G.9154/MT.4171: respectively, 7 and 6 possible syntypes, ex de Cort coll.

####### Type locality.

Switzerland: Andeer.

####### Taxonomic status.

Junior synonym of *Ariantaarbustorum* (Linnaeus, 1758).

####### Discussion.

The two available specimen lots were collected at the type locality Andeer and stem from the collection of Hugo de Cort. There is no indication that they came from Roffiaen’s collection, but the specimens are a good conchological match to Roffiaen’s brief description and figures (Roffiaen 1868: pl. 1, fig. 2a, b). Thus, we interpret the present specimens as possible syntypes.

This variety was described to denote specimens with an elongated spire, which gives the shell a more trochoid shape. As mentioned above for shell colouration, intraspecific variation in shell shape (including spire height) is common across members of *A.arbustorum* (e.g., Germain 1930; Baminger 1997). Thus, *Helixarbustorumtrochoidalis* is here considered synonymous with *A.arbustorum*.

## ﻿Conclusion

The present study brings to light the type specimens of nine out of the 14 new taxa described by Roffiaen (1868)—taxa that had nearly sunk into oblivion in most of the academic literature. This serendipitous finding of type specimens reaffirms the importance of maintaining museum collections, providing funding for digitization programs to uncover/recover such information and, therefore, make it available to the scientific community at large.

## Supplementary Material

XML Treatment for Paludina contecta var. emiliana

XML Treatment for Valvata colbeaui

XML Treatment for Lymnaea palustris var. fallaciosa

XML Treatment for Lymnaea palustris var. pellucida

XML Treatment for Lymnaea stagnalis var. productissima

XML Treatment for Lymnaea peregra var. pulchella

XML Treatment for Lymnaea truncatula var. subangulata

XML Treatment for Clausilia plicatula var. albinos

XML Treatment for Clausilia plicata var. elongata

XML Treatment for Clausilia weyersi

XML Treatment for Helix ruderata var. viridana

XML Treatment for Helix arbustorum var. icterica

XML Treatment for Helix arbustorum var. marmorata

XML Treatment for Helix arbustorum var. trochoidalis

## ﻿Additional information

### Author ORCIDs

Rodrigo Brincalepe Salvador https://orcid.org/0000-0002-4238-2276

Cedric d'Udekem d'Acoz https://orcid.org/0000-0002-7893-305X

Maxim V. Vinarski https://orcid.org/0000-0002-7644-4164

Yves Samyn https://orcid.org/0000-0002-1653-3018

Barbara Mizumo Tomotani https://orcid.org/0000-0002-8855-4803

### Data availability

All of the data that support the findings of this study are available in the main text or Supplementary Information.

## ﻿Appendix 1

**List of publications by Roffiaen**

The list below includes Roffiaen’s publications, as well as selected transcriptions and/or summaries of communications presented by him during the meetings of the Société malacologique de Belgique.

Roffiaen F (1865) Notes conchyoliogiques. [Translation to French of an article by A. Senoner.] Annales de la Société royale malacologique de Belgique 1: 15–21.

Roffiaen F (1867) Mollusques de l’Algérie déposés à Genck. Annales de la Société royale malacologique de Belgique 2: xxxv.

Roffiaen F (1867) Coquilles recueillies à Diepenbeek. Annales de la Société royale malacologique de Belgique 2: ·eu.

Roffiaen F (1867) *Helixhortensis* scalariforme à Tervueren. Annales de la Société royale malacologique de Belgique 2: xcvii.

Roffiaen F (1868) Mollusques terrestres et fluviatiles recueillis en Suisse. Annales de la Société malacologique de Belgique 3: 65–84.

Roffiaen F (1868) *Helixnemoralis* var. 19 à Bruxelles. Annales de la Société malacologique de Belgique 3: xxxii.

Roffiaen F (1868) Essais pour obtenir les *Helixscalariformes*. Annales de la Société malacologique de Belgique 3: lxxxii–lxxxiv.

Roffiaen F (1869) *Helixarbustorum* à Hastière. Annales de la Société malacologique de Belgique 4: lv.

Roffiaen F (1870) Quelques mollusques de Namur. Annales de la Société malacologique de Belgique 5: p. xvii.

Roffiaen F (1870) *Helixpulchella* au Mont-St-Bernard. Annales de la Société malacologique de Belgique 5: p. xvii–xviii.

Roffiaen F (1871) Bulimus acicula vivant à Yvoir. Annales de la Société malacologique de Belgique 6: lii.

Roffiaen F (1871) Coquilles recueillies à Hastière, Bouillon, et Chimay. Annales de la Société malacologique de Belgique 6: lvi.

Roffiaen F (1873) Liste de mollusques recueillis dans la vallée de l’Ourthe. Annales de la Société malacologique de Belgique 8: cxx–cxxi.

Roffiaen F (1874) Mollusques recueillis dans le Grand-Duché de Luxembourg. Annales de la Société malacologique de Belgique 9: clviii–clix.

Roffiaen F (1875) Mollusques des environs de Gand. Société malacologique de Belgique 10: xxxiii–xxxiv.

Roffiaen F (1875) *Helixhispida* senestre à Waulsort. Société malacologique de Belgique 10: lxvi.

Roffiaen F (1875) Mollusques recueillis aux environs de Gand. Annales de la Société malacologique de Belgique 10: lvi–lvii.

Roffiaen F (1875) Diverses coquilles recueillies à Waulsort. Annales de la Société malacologique de Belgique 10: lxvi.

Roffiaen F (1876) Mollusques recueillis aux environs de Gand. Annales de la Société malacologique de Belgique 11: xlix.

Roffiaen F (1877) Notes sur des mollusques terrestres et fluviatiles recueillis à Waulsort (1877). Annales de la Société malacologique de Belgique 12: lxxvi–lxxix.

Roffiaen F (1879) Mollusques recueillis en Suisse em 1879. Annales de la Société malacologique de Belgique 14: lxxxiv–lxxxv.

Roffiaen F (1881) Jules Colbeau et la Société Royale malacologique de Belgique. Annales de la Société royale malacologique de Belgique 16: i–xxxi.

Roffiaen F (1882) Assemblée Générale anuelle du 2 juillet 1882. Rapport du President. Annales de la Société royale malacologique de Belgique 17: 128–141.

Roffiaen F (1888) Quelques mollusques de Waulsort et d’Hastière. Annales de la Société royale malacologique de Belgique 23: lxxxvi– lxxxvii.)

Roffiaen F, Timmermans D (1867) Rapport sur le travail de J. Sauveur. Annales de la Société royale malacologique de Belgique 2: lxxxix–xcii.

## Supplementary materials

Supplementary material 1 CT scan snapshots illustrating the internal structures of the shell of *Clausiliaweyersi* Roffiaen, 1868 Data type pdf https://binary.pensoft.net/file/1335044This dataset is made available under the Open Database License (http://opendatacommons.org/licenses/odbl/1.0/). The Open Database License (ODbL) is a license agreement intended to allow users to freely share, modify, and use this Dataset while maintaining this same freedom for others, provided that the original source and author(s) are credited.Rodrigo Brincalepe Salvador, Cedric d'Udekem d'Acoz, Maxim V. Vinarski, Yves Samyn, Barbara Mizumo Tomotani
