# Supplementary material for: ﻿François Roffiaen's terrestrial and freshwater gastropod types in the collection of the Royal Belgian Institute of Natural Sciences
Source: Zookeys. 2025 May 21;1239:103–21. doi: 10.3897/zookeys.1239.150840 (PMC12120496; doi:10.3897/zookeys.1239.150840)
Supplement: Supplementary material 1 — CT scan snapshots illustrating the internal structures of the shell of Clausiliaweyersi Roffiaen, 1868 [file zookeys-1239-103_article-150840__-s001.pdf]

SUPPLEMENTARY FILE

This file contains supplementary material to the article "*François Roffiaen's terrestrial and freshwater gastropod types in the collection of the Royal Belgian Institute of Natural Sciences*" by R.B. Salvador, C.d'U. d'Acoz, M.V. Vinarski, Y. Samyn, and B.M. Tomotani.

Snapshots from the CT scan rendering of *Clausilia Weyersi* Roffiaen, 1868 (presently considered a junior synonym of *Laciniaria plicata plicata* (Draparnaud, 1801)), showing the shell's external surface and internal structures.

The scanned specimen belongs to lot RBINS I.G.9154/HIST.2486, which contains 29 potential syntypes (see main text for more info).

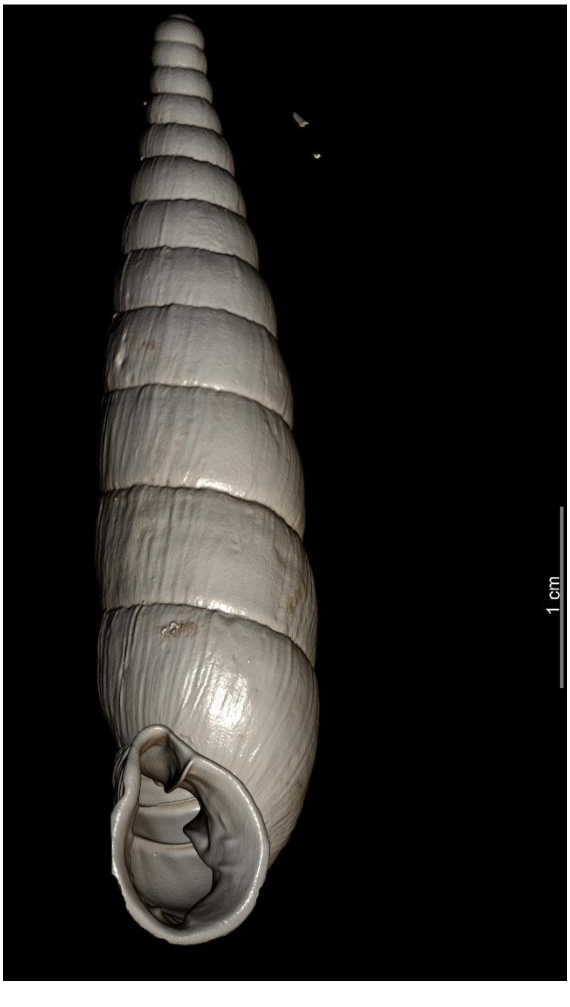

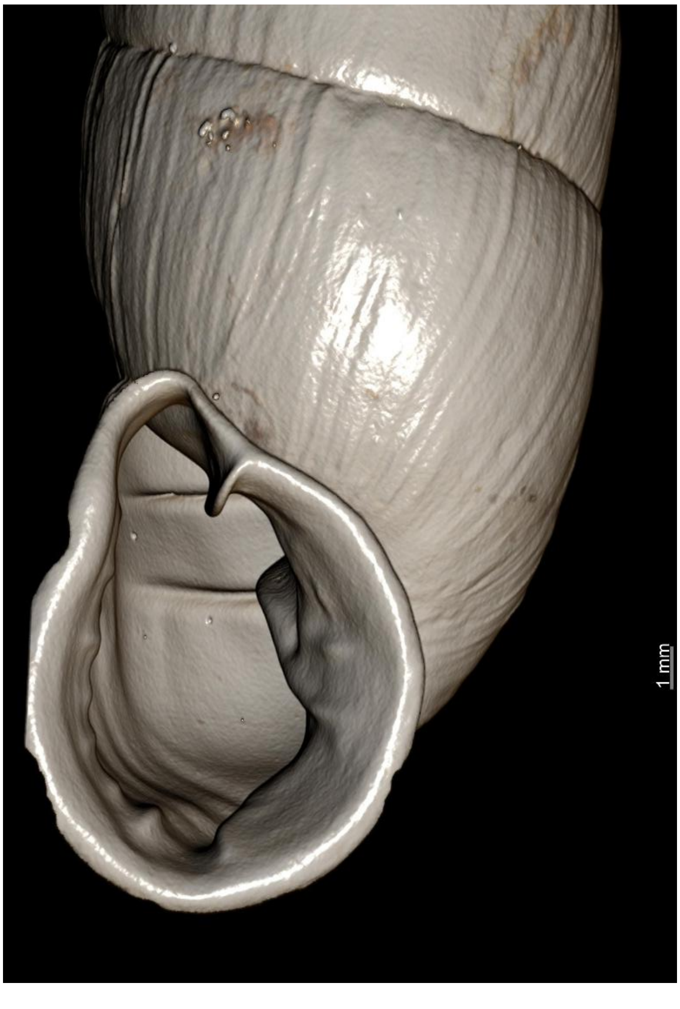

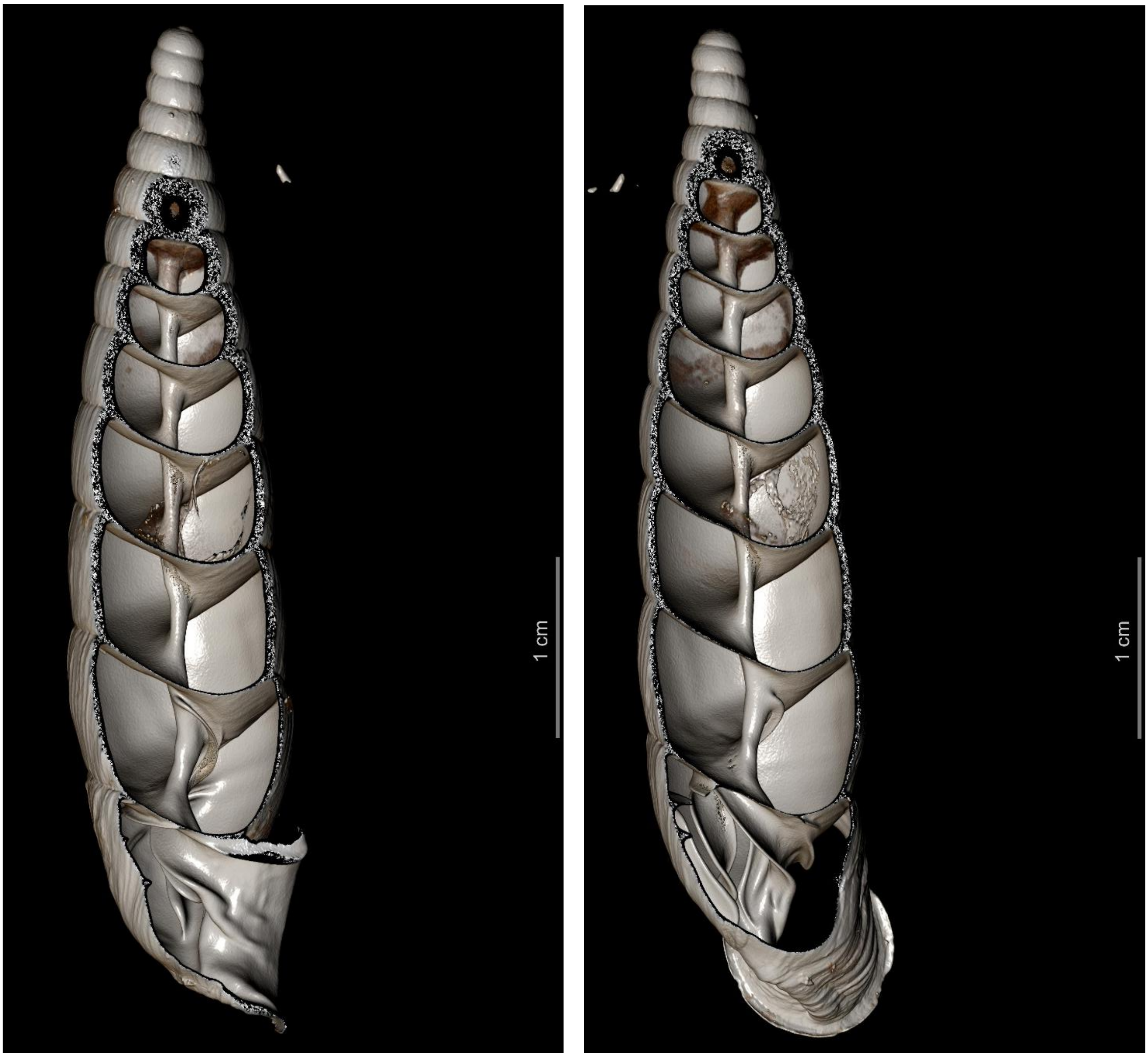
1 cm
1 cm

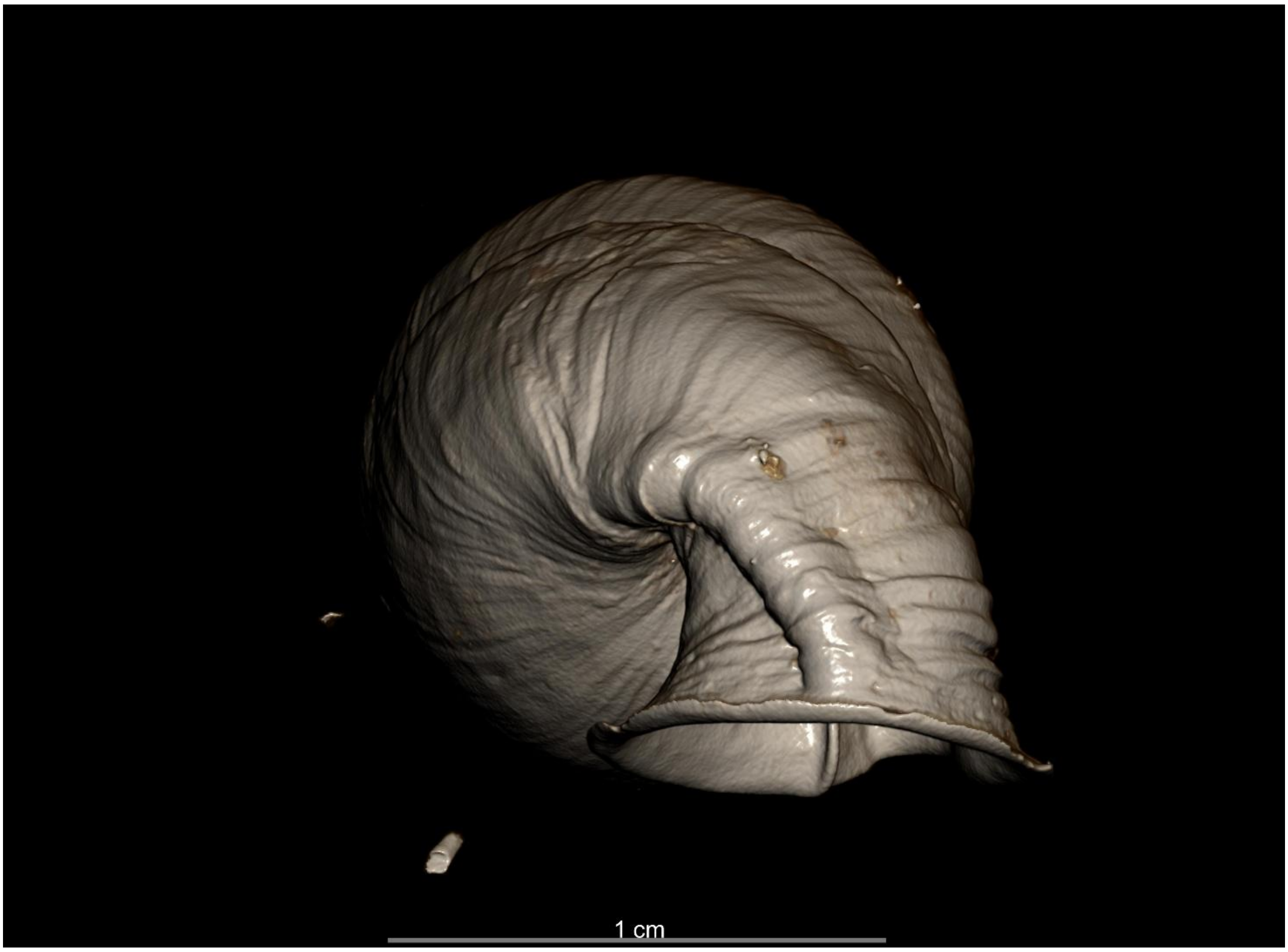
1 cm

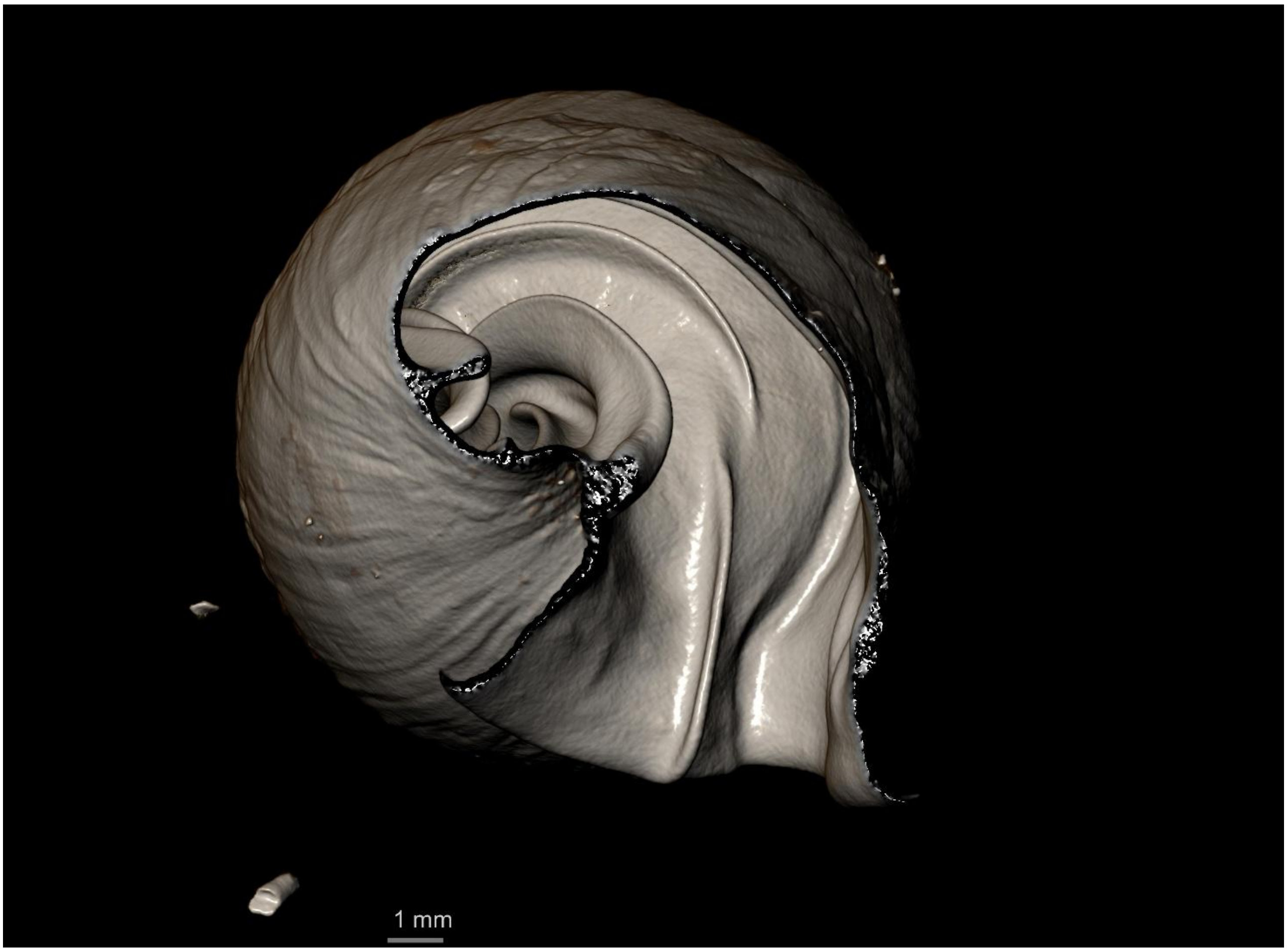
1 mm
